# The Global Health System: Lessons for a Stronger Institutional Framework

**DOI:** 10.1371/journal.pmed.1000193

**Published:** 2010-01-26

**Authors:** Suerie Moon, Nicole A. Szlezák, Catherine M. Michaud, Dean T. Jamison, Gerald T. Keusch, William C. Clark, Barry R. Bloom

**Affiliations:** 1 Sustainability Science Program, John F. Kennedy School of Government, Harvard University, Cambridge, Massachusetts, United States of America; 2 Harvard Initiative for Global Health, Harvard University, Cambridge, Massachusetts, United States of America; 3 Department of Global Health, University of Washington, Seattle, Washington, United States of America; 4 Global Health Initiative, Boston University, Boston, Massachusetts, United States of America; 5 Harvard School of Public Health, Boston, Massachusetts, United States of America; London School of Hygiene and Tropical Medicine, United Kingdom

## Abstract

In the last in a series of four articles highlighting the changing nature of global health institutions, Suerie Moon and colleagues propose future actions to strengthen these institutions.

*This is the fourth in a series of four articles that highlight the changing nature of global health institutions.*

## Introduction

The global health system is in a period of rapid transition, with an upsurge of funds and greater political recognition, a broader range of health challenges, many new actors, and the rules, norms and expectations that govern them in flux. The introductory article of this series (Szlezák et al. [1]) laid out some of the many challenges facing the global health system. This system is defined as the constellation of actors (individuals and/or organizations) “whose primary purpose is to promote, restore or maintain health [2]” and “the persistent and connected sets of rules (formal or informal), that prescribe behavioral roles, constrain activity, and shape expectation [3]” among these actors. The second article (Frenk [4]) defined the key attributes of national health systems as a core component of the global system. The third article (Keusch et al. [5]) analyzed the institutional evolution of one of the system's most important functions—the integration of research, development, and delivery.

This concluding article draws on the others in the series. It also draws from a year-long effort that included case studies, two international workshops of scholars and practitioners (further details at http://www.hks.harvard.edu/centers/cid/programs/sustsci/events/workshops/2008/institutions), and ongoing discussions by the authors, to summarize lessons learned and propose future actions to strengthen the system as a whole. The project used as a case study the global health system's evolving response to malaria. Nevertheless, the workshops and discussions that informed this analysis drew from a broader range of cases, and we believe lessons learned may usefully apply beyond malaria alone. Furthermore, while recognizing the many determinants of health and interlinkages between health and other issue areas such as trade and environment [6],[7], we limit our scrutiny here to the global health system.

The project concluded that an effective global health system must accomplish at least five core functions: agenda-setting; financing and resource allocation; research and development (R&D); implementation and delivery; and monitoring, evaluation, and learning. We discuss here ways to improve each of the five functional areas, consider the implications for the role of the World Health Organization (WHO), and make recommendations for future action.

## Key Functions of the Global Health System

### Agenda-Setting

In the past, global agenda-setting in health took place within the framework of the United Nations (UN)—primarily at WHO and the UN Childrens Fund (UNICEF)—with input from national governments and a few foundations. It was exemplified by iconic programs such as the eradication initiatives for malaria and smallpox in the 1950s–70s. Agenda-setting is well captured by a “punctuated equilibrium” model, in which long periods of relative stability in agendas are sporadically broken by sudden bursts of high-level attention in public and policy circles [8],[9]. Agendas may vary because of crises, such as natural disasters or epidemics, or from recognition of the human and economic costs of inaction, as with noncommunicable diseases [10]. History indicates that these episodes of high attention are fleeting; seizing these brief opportunities to produce lasting change usually requires adapting governance structures to accommodate new actors and interests [11].

Our case study of malaria found that, after undergoing a half-century of fluctuating global attention, malaria re-emerged on the global agenda in the late 1990s. Central to its reemergence was the creation of a novel global governance structure, the Roll Back Malaria (RBM) Partnership, launched by WHO. RBM now includes over 100 organizations including endemic country governments, donors, civil society organizations, the private sector, and academia.

Once an issue garners attention and attracts many new actors and activities, effective governance requires a process for setting an agenda for action *within* the issue area. Coordination is ultimately essential; however, as several experienced participants in our workshops pointed out, few organizations wish to be coordinated, because of the costs and loss of autonomy entailed. Thus coordination and some degree of harmonization of multiple independent activities are likely to emerge only after the construction of consensus on a widely shared set of rules, roles, and expectations. To get to this consensus, participants must share a clear set of goals and perceive the process as inclusive, transparent, technically credible, and fair.

Effective agenda-setting for action, when achieved, can provide a framework (albeit no guarantee) for coordination at global and national levels. The 2008 Global Malaria Action Plan, which was negotiated within the RBM framework, exemplifies how global agenda-setting for action within an issue area can be achieved [12]. A similar institution, the Stop TB Partnership, has also created a coordinating framework for tuberculosis control.

Underlying such institutional frameworks must be scientifically valid metrics, evidence of the problem's importance, and recognition that tools exist, however imperfect, that could improve health outcomes. Finally, the framework requires that the affected countries and the public, who are ultimately co-producers of health, be represented as key participants [4]. These partnerships, anchored by the legitimacy of the WHO, represent creative approaches to eliciting the broad participation necessary to construct widely accepted agendas and forge consensus at the global level.

### Financing and Resource Allocation

International financing and resource allocation for health in developing countries have long been subject to three fundamental questions [13]: (1) How should the priorities of donors be balanced with those of recipients? (2) How should resources be allocated to different diseases or issue areas? (3) How can sufficient investment into health, which has traditionally been underfunded relative to need, be ensured?

In the past, international resources for health flowed primarily through bi-/multilateral donors and WHO with only a few exceptions, e.g., the Rockefeller Foundation. Over the past decade a variety of actors, including philanthropists, advocacy groups, civil society, and public and private sector organizations, have catalyzed an unprecedented increase in the flow of international financing for health [14]. In the case of malaria, funding has increased tenfold [12]. The Global Fund to Fight AIDS, Tuberculosis and Malaria (GFATM) represents a model for enabling some coherence in the resource allocation process for its mandated diseases. The GFATM balances two often-competing objectives: providing reassurance to donors by only funding projects adhering to international “best practices,” and demanding that country applications demonstrate meaningful and widespread national ownership [15]. By unifying multiple funding streams and oversight processes, the GFATM also tries to lighten the burden put on national health systems by reporting requirements and lack of coordination among multiple donors—an issue exacerbated by the recent increase in players in the global health system.

While the upsurge of financing for malaria is welcome, it also points to a current governance gap in the overall system: there are no clear norms for how resources should be allocated across different health needs. The Global Burden of Disease and Disease Control Priorities Projects have provided country estimates of years of healthy life lost to illness and injury, identified major risks, and estimated the cost-effectiveness of interventions [16],[17]. However, widely accepted principles on how to translate these figures into resource allocation decisions are lacking. Major discrepancies exist between resources provided for specific diseases and their relative burden, e.g., HIV/AIDS versus chronic diseases, and between the burdens of disease within specific countries and their ability to attract resources to address them [18].

A related question is how to increase funding levels further to meet the full spectrum of global health needs, and how to sustain those levels in the long run. Especially in difficult economic times, it is critical to ensure continuity by insulating finance arrangements to the greatest extent possible [19]. In all World Bank regions, external development assistance represents less than 3% of total health spending, with the exception of sub-Saharan Africa where it accounts for 21% [20]. However, international financing is critical for providing global public goods and for the lowest-income countries that rely on aid to meet basic health needs. Financial fluctuations can be disruptive in all countries, but for the poorest, a sudden drop in aid can be devastating. The long-term sustainability of financing will rest on three elements: (1) demonstrating results; (2) making financing arrangements more politically acceptable by mobilizing more resources from middle-income countries; and (3) developing innovative financing mechanisms that are less vulnerable to politicized budgeting processes. One such innovative model is UNITAID, which purchases health products for use primarily in low-income countries and is funded through national airline taxes. Of the 29 committed UNITAID donors, three-quarters are low- or middle-income countries, emphasizing the idea that all nations—even the poorest—can contribute to sustainable global health finance [21].

### Research and Development

In the past, health technologies such as drugs, vaccines, and diagnostics were developed primarily by and for populations in the industrialized world. Today there is increasing evidence of contributions from the South to global health research. Investments in human capacity that began in the 1970s are now bearing fruit as scientists from Africa, Asia, and Latin America take a key role in advancing research, as in the case of malaria [5]. After a period of neglect, there is now a resurgence of R&D aimed specifically at developing new tools for the health needs of developing countries. Since traditional market incentives such as the patent system are unlikely to generate the necessary innovation, much of malaria R&D is now taking place through public–private product development partnerships (PDPs), which receive significant philanthropic, public, and private investments [22]. In contrast to classic private-sector product development, however, the PDPs have an explicit objective of jointly achieving affordability and innovation suited to developing country contexts. PDPs are redefining the roles of public and private sectors and promoting new expectations for the development of health technologies as global public goods.

Experience with malaria offers several lessons for R&D in other health areas. First, investments in human capital are essential but take many years to bear fruit. Here the long-term commitment of the UNICEF/UNDP/World Bank/WHO Special Programme for Research and Training in Tropical Diseases (TDR) and the recent £30 million commitment from the Wellcome Trust for research capacity building in Africa should be noted [23]. Nevertheless, greater training in laboratory sciences, health economics, management, program evaluation, and implementation research are clearly needed. Capacity-building of developing country researchers and research organizations (e.g., universities, public research institutes) should receive greater emphasis and be scaled up today. Second, considerations of access to products should be built into R&D processes from their inception. The WHO Global Strategy and Plan of Action on Public Health, Innovation and Intellectual Property, approved at the 2008 World Health Assembly (WHA), is an important contribution to rethinking the governance of the R&D system, and merits the constructive engagement of all concerned parties [24].

### Implementation and Delivery

As the essential link between global actors and local populations, national health systems are a critical part of the global health system. Health systems must accomplish multiple challenging tasks. These include: providing preventive and primary care services; developing a health workforce; devising equitable financing arrangements; regulating the private sector; and leveraging vertical programs (such as malaria control) to strengthen, rather than distort, the overall health system (“diagonalization” [25]) ([4]).

Health system performance varies widely but the reasons for this variation remain poorly understood. For example, Eritrea, Ethiopia and Rwanda have reduced malaria-related morbidity and mortality dramatically [26],[27]. Eritrea, for one, credits the RBM strategy and community health workers as key components of their approach [28]. However, it is unclear why, largely using the same strategies recommended to all endemic countries, others were less successful. Recent analyses of health systems performance point to leadership, community involvement, district-level focus, use of data to set priorities and track progress, and prioritizing equitable access as key factors that have enabled significant improvements in health outcomes in some countries [4],[29]. Even when public sector delivery capacity is weak, some countries have still managed to expand primary health care coverage and improve childhood survival by engaging the private for-profit as well as nonprofit sectors [29]. These non-state actors can energize national health systems by sharing knowledge of how better to achieve efficiency, outreach, and user satisfaction. A comprehensive operational and policy research agenda is needed to understand fully which policies and practices best strengthen national health systems [30],[31].

### Monitoring, Evaluation, and Learning

Reliable information on the impact of health programs is critical to setting priorities, measuring efficacy, and maintaining global support for any intervention. Yet the global health system currently poorly manages monitoring and evaluation (M&E). There is no consensus on key questions regarding who should be responsible for M&E, how it should be carried out, how available the information should be, and how it should be used. National and subnational organizations for conducting M&E and promoting critical learning are relatively weak, and incentives for strengthening them are almost nonexistent.

For example, there remain enormous gaps in knowledge about malaria. Precise annual and seasonal malaria incidence and mortality data, or the percentage of children with fevers that actually have malaria, are unavailable in most endemic countries and districts [28],[32]–[34]. How to mobilize communities to make full use of bed nets, artemisinin combination therapies (ACTs), and indoor spraying remain critical research issues. With increasing funds being expended on programs, it is shortsighted that so little is spent on operational research, on learning what works in specific contexts and how best to engage communities to use the tools available [35]. An essential step toward sufficient investment in M&E is to acknowledge and plan for its costs, both in dollars and, more importantly, in the limited time available from experienced managers and researchers. This research is vital, yet ironically it is too rarely funded by major donors nor requested by implementing countries.

M&E should be an integral part of all program planning, yet it is too often an afterthought. Furthermore, effective M&E of programs and interventions, as well as learning from experience, requires that M&E efforts achieve technical *credibility*, maintain *legitimacy* (i.e., general acceptance of their authority), and produce knowledge that is *salient* for end-users. These three objectives often compete directly with one another [36]. For example, there is often tension between the goal of producing data for internal learning and that of monitoring by outside parties (e.g., higher-level officials or donors). When evaluation data are linked to funding, as in currently favored “performance-based funding,” the accuracy of the data provided may diminish. It may be necessary to create institutional “safe spaces” where failures can be divulged to encourage genuine learning rather than used to assign blame for underperformance [37],[38].

Similarly, at the global level, all countries provide health statistics to WHO, but the quality and validity of these data vary greatly. Yet WHO is a producer, repository, and evaluator of evidence, and simultaneously a political organization representing 193 countries, and so it will be particularly difficult for the organization to meet the demands for saliency, credibility, and legitimacy. Academic researchers, who also play a critical role in M&E, face a contrasting set of difficulties. While they may achieve technical credibility, it is more challenging to achieve political legitimacy and ensure that the knowledge produced is salient to end users and policy-makers.

Several new organizations are addressing some of these issues. For example, the Institute for Health Metrics and Evaluation [39] and the WHO-hosted Health Metrics Network [40], both funded in part by the Bill & Melinda Gates Foundation, were recently created to develop methods to acquire, analyze and disseminate relevant and reliable information on burden of disease and global health. There are promising models, some country-based such as the India Program Evaluation Network coming out of the International Clinical Epidemiology Network (INCLEN) [41] and the India network, IndiaCLEN [42], others international, such as the Southern-led INDEPTH epidemiological network [43] and the Cochrane Collaboration [44]. The challenge is not only to secure the evidence, but also to have the political and procedural legitimacy that, to date, few organizations other than the UN agencies have been chartered to provide.

## The Role of WHO

Cutting across these five core functions is the question of how changes in the global health system redefine the role of WHO. WHO is facing “an urgent need to define and assert a clear and effective role for itself, as never before” [45]. There are at least three key roles that we believe only WHO can fulfill and therefore must do well. The first is global stewardship, i.e. identifying needs to be met and taking a leadership role in setting global norms. Second is as a provider of operational support to countries: WHO has a unique capacity to engage the best experts worldwide, which should enable it to provide technical assistance to governments through normative guidelines and recommendations reflecting best evidence and practice. To retain the legitimacy to do so, it must maintain the highest technical and ethical standards [46]. The third is its special role in governance: as the major global *intergovernmental* health organization, WHO has a unique convening power and mandate for decision-making on major health-related issues. Its governing body, the World Health Assembly, with its 193 Ministers of Health, provides WHO its singular legitimacy to carry out these mandated roles of stewardship, country support, and governance, albeit with a high degree of bureaucratization and politicization. Yet WHO's regular budget resources are remarkably limited. For the 2006–7 biennium, the formal budget assessed on countries was less than $1 billion (with voluntary contributions the total budget was just over $3 billion); the following period, three-fourths of the budget was allocated to the regions [47]. This excessive budgetary decentralization undermines WHO's capacity to deliver the global public goods demanded of it.

Inadequate levels of core funding have resulted in predictable consequences for performance. For example, WHO's malaria program has experienced a number of difficulties in recent years, and while inadequate funding is not the only cause it is an important one. When WHO fails to lead, new global partnerships such as RBM (originally created by WHO's Director-General) have stepped in, received external funding, and, to an extent, WHO has been marginalized. For WHO to fulfill the key roles for which it is uniquely charged, it will need strong leadership, strengthened technical expertise, and clearer focus. The current economic crisis provides an opportunity for WHO to redefine and strengthen its core functions, recognize what it cannot do well, and delegate to or partner with other organizations. If it can define its core functions and strategic role credibly, the organization will justify—and perhaps be more likely to receive—the greater resources it will need to fulfill its central global mission.

## Lessons and Future Needs

Several general lessons have emerged from our study of institutions in the global health system.

- In the present complex global environment no single actor can or should set the agenda for action. Global partnerships similar to those that have transformed malaria and the infectious disease agenda will be needed to mobilize resources for other health problems, such as chronic diseases. An example is the new Global Alliance on Chronic Diseases [48]. Broad-based, participatory processes for agenda setting, anchored by WHO's global political legitimacy, will be required to define priorities, avoid unnecessary duplication, and share knowledge. There is clearly a tension between WHO as an *intergovernmental* organization and WHO as a partner in multiple partnerships where it must share power with a broader set of *nongovernmental* actors including civil society organizations, foundations, and the private sector. Widely accepted procedural principles including transparency, broad participation, integrity of data, and equity should be adopted to construct the consensus necessary for effective coordination.
- Sustainability depends on strengthening national health systems, which are the essential link between global knowledge and best practices, and local health needs and impact. Disease-specific international funding also has much to contribute. But it can distort national priorities, pull resources from less-popular programs, and ultimately undermine the overall performance of the health system [49]. Country experts are often in a better position to set priorities than outside consultants. Donors should allow greater flexibility for recipient countries to direct a portion of received funds beyond narrow programmatic interventions to strengthening national health systems. This will require the development of clearly defined goals and performance indicators for key functions of health systems such as service provision, research, health worker development, and equity of access.
- Ironically, the proliferation of global actors threatens to weaken health systems by placing additional reporting burdens on already thinly stretched health ministries [49]. By channeling multiple funding streams into a single source for HIV/AIDS, tuberculosis, and malaria, the GFATM offers an instructive example of how to distribute the resources of various donors in a way that is sensitive to national health systems' priorities and constraints. As new global health initiatives arise to address the wave of emerging health challenges, the global health system should identify and adopt analogous ways to streamline reporting and, more generally, to minimize the additional transaction costs put on countries.
- Systematic investment in creating new and improving existing M&E programs should become second nature for all global health activities. The global health system has two important functions to fulfill. First, it needs to set the tone and actively foster the idea that M&E is crucial to global health. Second, it needs to support the systematic exchange, coordination, and streamlining of M&E efforts. Over time, this investment will contribute to building robust M&E systems and to generating reliable, comparable data to inform action.
- There is compelling evidence that long-term investments in education and training at many levels (e.g., national, provincial, district) can result in large payoffs for improved health [5],[50]. The global health system should prioritize additional investments in longer-term, multidisciplinary education and training for leadership in the complex public health, medical, management, economic, education, communications, and policy aspects of health systems, and in the functioning of health systems overall.
- Finally, it will be critical to support research that provides the evidence and knowledge bases for prioritization, resource allocation, and the development and evaluation of new tools and interventions. In particular, operational research will be crucial to learning how to use the tools that are available, take them to scale, and engage populations to become co-producers of health rather than passive recipients of services. More broadly, research should be promoted to understand variation in the performance of different national health systems, and thus to identify system designs that can be adapted to local circumstances to help translate global aspirations into meaningful impact on people's lives.

## Abbreviations

ACT: artemisinin combination therapy
GFATM: Global Fund to Fight AIDS, Tuberculosis and Malaria
INCLEN: International Clinical Epidemiology Network
M&E: monitoring and evaluation
PDP: public–private product development partnership
R&D: research and development, RBM, Roll Back Malaria
TDR: Special Programme for Research and Training in Tropical Diseases
UN: United Nations
UNICEF: United Nations Children's Fund
WHA: World Health Assembly
WHO: World Health Organization
